# Characterization of Magnetron Sputtered BiTe-Based Thermoelectric Thin Films

**DOI:** 10.3390/nano13010208

**Published:** 2023-01-03

**Authors:** Zhenxue Zhang, Mikdat Gurtaran, Xiaoying Li, Hio-Ieng Un, Yi Qin, Hanshan Dong

**Affiliations:** 1 School of Metallurgy and Materials, The University of Birmingham, Birmingham B15 2TT, UK; 2 Cavendish Laboratory, University of Cambridge, Cambridge CB3 0HE, UK; 3 Design, Manufacturing and Engineering Management, University of Strathclyde, Glasgow G1 1XQ, UK

**Keywords:** thermoelectric, magnetron sputtering, BiTe, thin film

## Abstract

Thermoelectric (TE) technology attracts much attention due to the fact it can convert thermal energy into electricity and vice versa. Thin-film TE materials can be synthesized on different kinds of substrates, which offer the possibility of the control of microstructure and composition to higher TE power, as well as the development of novel TE devices meeting flexible and miniature requirements. In this work, we use magnetron sputtering to deposit N-type and P-type BiTe-based thin films on silicon, glass, and Kapton HN polyimide foil. Their morphology, microstructure, and phase constituents are studied by SEM/EDX, XRD, and TEM. The electrical conductivity, thermal conductivity, and Seebeck coefficient of the thin film are measured by a special in-plane advanced test system. The output of electrical power (open-circuit voltage and electric current) of the thin film is measured by an in-house apparatus at different temperature gradient. The impact of deposition parameters and the thickness, width, and length of the thin film on the power output are also investigated for optimizing the thin-film flexible TE device to harvest thermal energy.

## 1. Introduction

In the past decades, extensive research on renewable energy has witnessed a significant advance in harvesting thermal energy into electricity via different pathways to maximize the Seebeck coefficient (S), the electrical conductivity (σ), and the reduction in the thermal conductivity (κ) [1]. The progress of the conventional heavy-atom-doped semiconductors, such as Bi_2_Te_3_, PbTe, Si–Ge, and Te–Ag–Ge–Sb, has resulted in increased thermoelectric figure of merit (ZT), which makes the technology more efficient at producing power [2]. To overcome the deficit of rare elements such as tellurium and avoid hazardous elements such as lead, alternative materials, such as bismuth sulfide (Bi_2_S_3_) [3], tin selenide (SnSe) [4], and magnesium-silicon-based new materials [5], have attracted much attention, and higher performance reports emerge regularly. However, the properties of bulk TE materials are determined primarily by composition and microstructure, which limits the usage of thermoelectric energy conversion to niche applications. Furthermore, bulk TE materials are limited by shape and size, which restricts their application in the area of intelligent and flexible TE devices. In contrast, thin-film technology has an advantage in controlling the composition, thickness, and microstructure, and higher TE power can be realized by quantum effects and band-gap engineering using reduced-dimensionality materials [6]. The nanosized structure results in the presence of a large number of interfaces (grain boundaries), which may effectively scatter phonons and thus reduce the thermal conductivity considerably and lead to a substantial increase in thermopower [7]. In the meantime, diversified substrates, for example, polymers, can be used to meet the flexible, lightweight, and miniature requirements [8].

Different film-growth methods based on molecular-beam epitaxy (MBE) [9], metalorganic chemical vapor deposition (MOCVD) [10], and flash evaporation [11] have been used to grow single layers and superlattices on various substrates. Evaporation is not appropriate for compound film deposition such as Bismuth telluride as the vapor pressure of Te is as much as 104 times higher than that of Bi and Sb at the melting points. The CVD technique often needs a higher temperature, while MBE has a very low deposition rate, which restricts its application. Magnetron sputtering is a versatile technique with a high deposition efficiency, and it can deposit conductible materials as well as insulative materials by using different powers such as direct current (DC), radio frequency (RF), or pulsed DC power. Therefore, the technique has stimulated the abundant interest of different groups to study various materials for high power output, as shown in Table 1.

In this paper, we used an unbalanced close-field magnetron sputtering ion plating technique to deposit nano-structured N-type and P-type BiTe-based thin films on silicon, glass, and Kapton HN polyimide foil. By adjusting the deposition parameters to modify the morphology, microstructure, and phase constituents, we have managed to optimize their thermoelectric properties. We have tested the electrical conductivity, thermal conductivity, and Seebeck coefficient of the sputter-deposited thin films using a dedicated in-plane test equipment. We have also used a home-designed device to examine the output of the electrical power (open-circuit voltage and electric current) of the thin film on the Kapton polymer substrate to optimize the deposition parameters and the thickness, width, and length of the thin film in order to design a device on flexible substrate. Clearly, nano-structuring materials become indispensable in the studies of thermoelectric function in consumer electronics [32], and this work offers the insights into the fabrication of the nanoscale thin-film thermoelectric flexible device.

## 2. Materials and Methods

### 2.1. Thin-Film Fabrication

In this work, the 99.99% purity of targets with a nominal composition of Bi_2_Te_3_Se (N-type) and BiSb_2_Te_3_ (P-type) plates in the size of 200 mm × 100 mm × 5 mm were bonded on a rectangle copper back plate by TEER Coatings Ltd. (UK). The thin film was deposited on glass, silicon wafer, special chip, and Kapton polymer substrates in a 4-target Teer Coating Ltd. closed field unbalanced magnetron sputtering ion plater in the direct current mode. A set of films were deposited with currents of 0.3 A, 0.4 A, 0.45 A, 0.5 A, and 0.6 A at various times. Detailed sample codes to corresponding coating conditions are listed in Table 2. The thickness was measured on the thin film deposited on the glass slide.

### 2.2. Characterisation and Properties Measurement

A Joel 7000 scanning electron microscope (SEM) equipped with an Oxford Inca energy dispersive X-ray spectroscopy (EDX) detector was used to inspect the surface morphology, cross-sectional microstructure, and elemental information of the thin film. An Inel EQUINOX 3000 (2 C) X-ray diffraction (XRD) using Cu-Kα radiation (λ = 0.154056 nm) was used to scan the surface from 20.01 and up to 80° with a step size of 0.02° and counting times of 2 s/step to examine the surface phase constituents. 300-square mesh copper TEM grids (Agar scientific, Essex, UK) were clipped to a plate, which was put into the PVD chamber to collect the coatings along the edge of the grids. A transition electron microscope (TEM) microstructure characterization was carried out by Jeol 2100 TEM.

**Figure 1:**
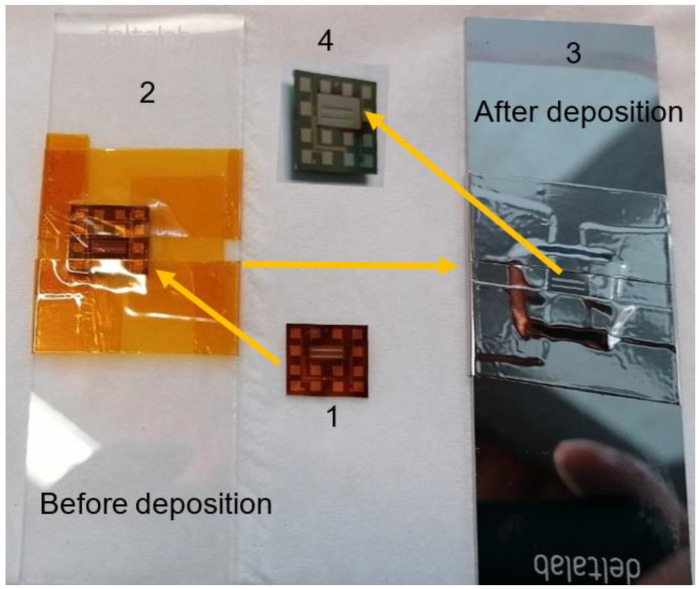
The preparation of thin film on chip: 1, the chip; 2, chip with attached shadow masks; 3, chips after deposition; and 4, chip with defined thin film ready for test.

An advanced platform for the in-plane ZT measurement was used to measure the thermal electric properties of the thin film. The film was deposited on a special ZT Al_2_O_3_ (ALD) Au contact chips for Linseis TFA supplied by SemiMetrics Limited (1 in Figure 1). The chip combines two suspended membrane setups, based on the Völklein geometry for in-plane thermal conductivity measurements with a 4-point Van-der-Pauw measurement setup for the determination of the electrical transport properties [33]. The chip was first covered by a metal mask or tape, and film was deposited on specified area as shown in Figure 1. After deposition, the mask and tape were removed and the chip (4) was ready for the test. The Seebeck coefficient is measured using a partly passivated resistance thermometer, located on the bigger membrane. Electrical conductivity, Seebeck coefficient, and thermal conductivity were measured at the same time within a temperature range between −150 °C and 250 °C.

### 2.3. Output Mesurement of the Active Thin Layers

**Figure 2:**
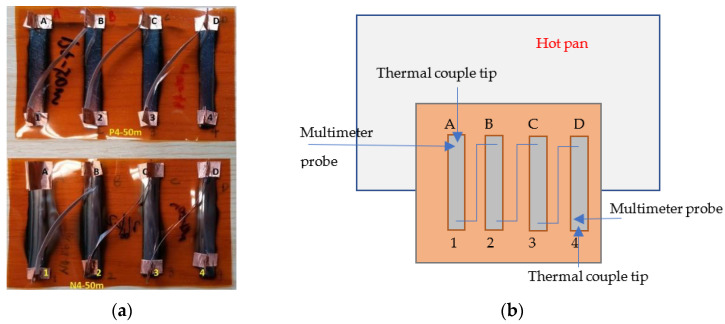
Setup for the measurement of the power output of thin film on Kapton substrate: (**a**) the film strips on Kapton substrate; (**b**) the sketch of the Kapton on a hot pan for test.

After evaluation, thin film strips with different widths and thicknesses of N-type and P-type layers were deposited on the Kapton substrate to assess their power output, as shown in Figure 2a. High-temperature conductive copper tape was used to link the strip and create connective points. One side of the strip (i.e., ABCD in Figure 2a) was put on a hot plate, which act as the heating source. The other side of the strip was left outside of the pan and cooled by naturally flowing air. Two thermal couple tips were buried under the copper tape connecting points at the hot and cold sides separately to monitor the temperature difference. One extra thermal couple tip was put on the hot pan to monitor the temperature of the heating. Two multi-meters were used to monitor the open circuit voltage/current and electrical resistance of different links such as A-1, A-4, or B-2, to assess the output of different configurations of the active layer strips. The temperature of the hot pan can be raised to 200 °C; accordingly, the tip on the active layer is about 100 °C, which can generate a temperature difference up to 80 °C. A schematic test apparatus is shown in Figure 2b.

## 3. Results and Discussion

### 3.1. Microstructure and Phase Constituents of the Thin Films

**Figure 3:**
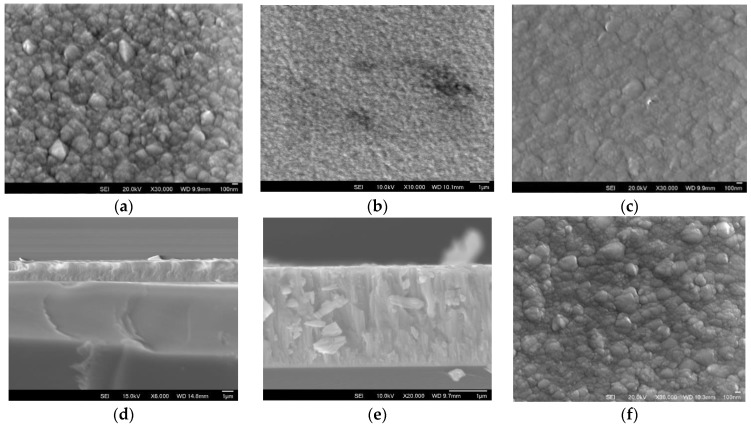
P-type BiSb_2_Te_3_ thin film deposited on silicon wafer: (**a**,**d**) P4, (**b**,**e**) P45, (**c**) P5, and (**f**) P6.

The surface morphology and cross-sectional microstructure of the P-type thin film deposited on silicon wafer with different target currents has been assessed by SEM (Figure 3). Crystallised clusters were formed on the surface after deposition, and the size only slightly changed with the increased current from 0.4 A to 0.6 A (Figure 3a–c,f). A dense fine columnar structure was formed, as seen in the cross-sectional image of P4 and P45 in Figure 3d,e. The composition of the thin film was identified by EDX, and the atomic composition ration of Bi:Sb:Te is about 2:3:4.

As shown in Table 2, both the N-type and P-type materials have a higher sputter rate, and it took only 40 min at 0.4 A to obtain a 2.0 µm thick N-type thin film. The deposition rate for P-type was slightly slower, as it took 50 min at 0.4 A to obtain only a 1.8 μm thick film. When the current was increased, the deposition was faster, but a higher current than 0.6A led to a spewing of material from the target, which adversely affected the film deposition, resulting in large particles on the surface, as evidenced in Figure 3f.

**Figure 4:**
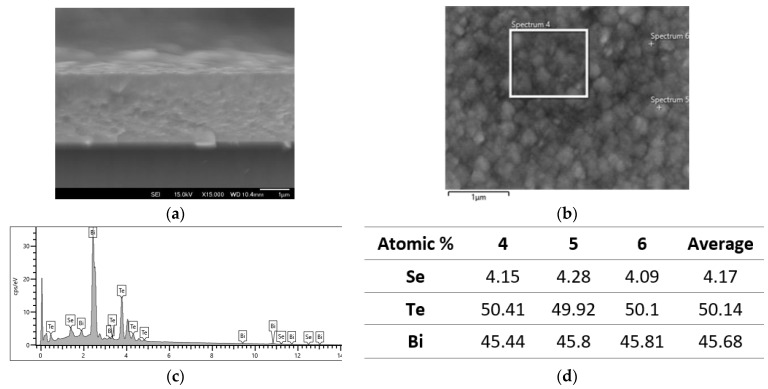
N-type (N4) Bi_2_Te_3_Se thin film deposited at 0.4A for 40 min: (**a**) cross-section; (**b**) surface; (**c**) EDX spectrum of Zone 4 in b; and (**d**) atomic composition of the film.

Typical cross-sectional and surface morphology images of the N-type layer on the silicon wafer are shown in Figure 4a,b. The composition of the thin film was identified by EDX, and the atomic composition was calculated as demonstrated in Figure 4c,d.

**Figure 5:**
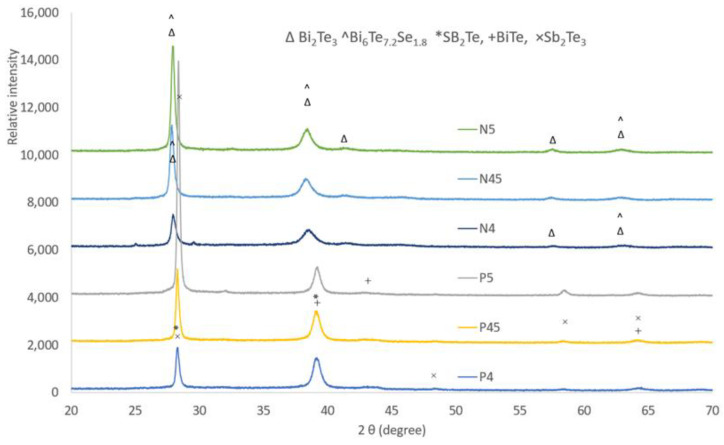
XRD comparison of different N-type and P-type layers on silicon substrate.

**Figure 6:**
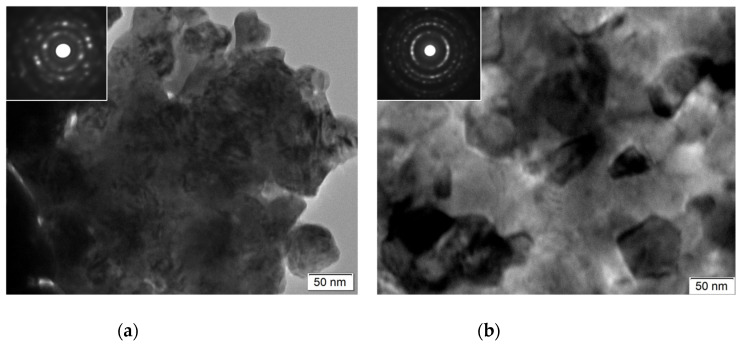
TEM microstructure and corresponding SAD patterns of (**a**) N5 and (**b**) P4.

Analyzing XRD patterns taken from the N-type and P-type thin films revealed similar phase constituents for each type film, and the representatives of the patterns are shown in (Figure 5). It can be seen that the N-type thin film consists of phases of Bi_2_Te_3_ (PDF001-085-0439) and Bi_6_Te_7.2_ Se_1.8_ (PDF096-0151-1977), while the P-type thin film is composed of three phases: Sb_2_Te (PDF0001-0080-1722), BiTe (PDF0001-0089-4303, and Sb_2_Te_3_ (PDF0000-0015-0874). For the N-type thin film, the grain size (Bi_2_Te_3_ at 2θ = 27.89°) increased with the deposition current, for example, it was about 28 nm for N4 as calculated using Scherrer’s equation, while it grew to about 45 nm for N5. Similarly, for the P-type film, the crystalline size (SB_2_Te_3_ at 2θ = 28.33°) increased from about 35 nm (P4) to about 53 nm (P5). TEM observation revealed equiaxed nano-grains with dimeters of 40–70 nm, as shown in Figure 6. SAD patterns taken from both P-type and N-type thin films featured ring-patterns, characteristic of nano-structured grains. The indexing of the d-spacing values calculated from the SAD patterns confirmed the phase composition results of the XRD analysis, shown in Figure 5.

### 3.2. Thermoelectrical Properties of the Thin Films

Thermoelectric properties measurements of the N-type and P-type thin films within the temperature range from −150 °C to 230 °C were measured simultaneously in an advanced measurement platform.

**Figure 7:**
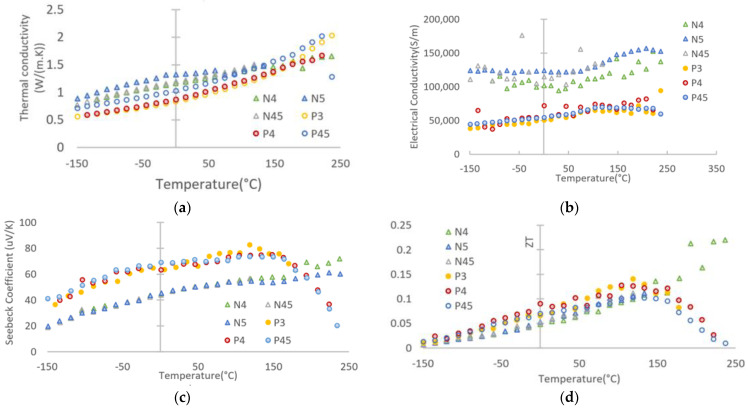
Thermal electric property measurements of the N-type and P-type thin films within the temperature range −150 °C to 230 °C: (**a**) thermal conductivity; (**b**) electrical conductivity; (**c**) absolute value change of Seebeck coefficient; and (**d**) calculated ZT value.

For all of the thin films, the thermal conductivity increased with the temperature, as shown in Figure 7a. Generally, the N-type layer has a higher thermal conductivity value in comparison with the P-type layer at a lower temperature range (<150 °C); this might be due to the grain-size effect as the presence of a large number of interfaces (grain boundaries), which may effectively scatter phonons while exerting a minimal effect on the transport of charge carriers [7]. In the meantime, the electrical conductivity of the N-type layers is significantly higher than those of the P-type layer, and it decreased slightly with the temperature increment from −150 °C to about 50 °C and then increased again till the end of the test (Figure 7b).

For the three P-type thin films, the electrical conductivity maintained the trend of climbing with an elevated temperature. The Seebeck coefficient of the P-type layers improved with temperature till about 140 °C and was superior to the N-type layer; however, it dropped gradually to a level lower than that of the N-type layer for further rising temperature (Figure 7c). Meanwhile, the Seebeck coefficient of N-type layers grew steadily with the temperature, which performed similarly to the N-type Bi_2_Te_3_ layer prepared at 300 C via DC magnetron sputtering [34]. As shown in Figure 7d, the calculated ZT value has a similar trend to the Seebeck coefficient, especially for the P-type layer. The ZT value change is generally higher than an 84 nm Bi_87_Sb_13_ thin film in Linseis’s work [33]. However, sample N4 performed the best at higher temperatures (>140 °C).

### 3.3. Output Test of the Thin Film

**Figure 8:**
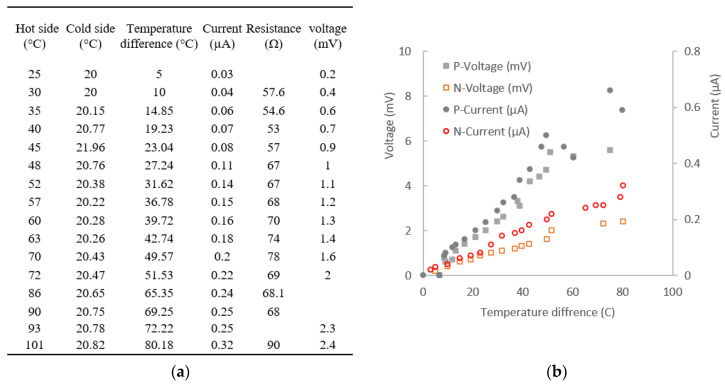
The output of thin film for both P-type (P4-G) and N-type (N4-G) layer on glass slide: (**a**) detail of the temperature change and related power output and resistance change; (**b**) the change of open circuit voltage and current against temperature difference.

To assess the capability of the thin film in generating power, a thin film of the active layer was deposited on a glass slide of 26 × 76 mm, and the end of two sides was wrapped with copper tape to extract the energy. Part of the glass slide was put on a heating pan as a hot part, and the other side left outside the pan was cooled by air act as the cool side. When the heater was turned on, the temperature difference between the hot side and cold side produced voltage and current, which were recorded by a multi-meter, as detailed in Figure 8a. The voltage increased with the growing temperature difference, as did the electric current (Figure 8b). This is in agreement with Francioso’s finding on RF-magnetron-sputtered SB_2_Te_3_ and Bi_2_Te_3_ thin film: the open circuit voltage and electrical power increase with increasing ∆T [35]. However, they reported a maximum output at ∆T = 40 °C, and we found the open circuit voltage increased further with the difference of temperature up to 80 °C. The resistance of the thin N-type film increased slightly with the increment in temperature. A similar change was observed on the P-type thin film on glass, and the open circuit voltage and current are higher for the P-type layer than those of the N-type layer. A follow-on test of thin film on the Kapton substrate shows a similar result—that the P-type layer has a higher open-circuit voltage and current than the N-type layer.

**Figure 9:**
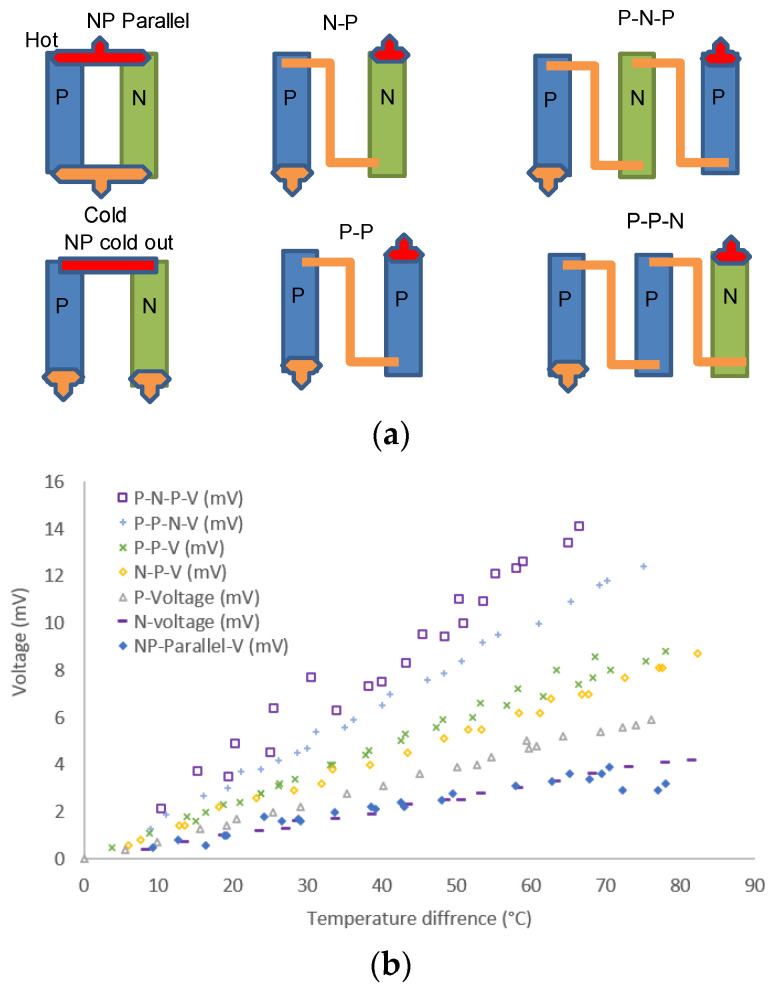
(**a**) Configuration of P-type and N-type layer (12 mm × 30 mm) on Kapton substrate; (**b**) open-circuit voltage–output comparison.

A different combination of the P-type and N-type layer (12 mm × 40 mm) on separated Kapton substrates was tested to investigate the effect of the power generation in different configurations such as P-N, P-P, N-N, PNP, PPN, etc. (Figure 9a). It was found that the hot-cold-hot-cold…series link generates most of the power; however, links such as hot side (N strip)–cold side (N strip)–cold side (P strip)–hot side (P strip) generate less power even less than one single P or N strip, as evidenced in Figure 9b.

**Figure 10:**
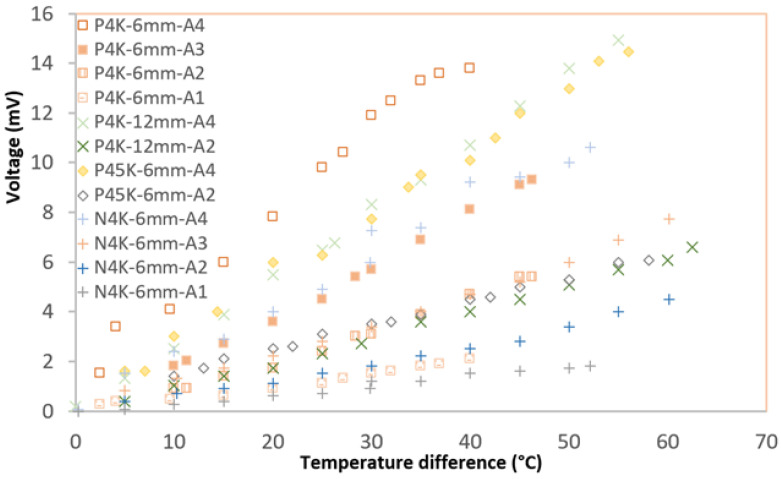
Open circuit voltage output of P-type layer with different widths (P4k-6 mm and P4k-12 mm) and thicknesses (P4k:2 μm and P45k:2.5 μm) on Kapton substrate.

After testing different configurations of P-type and/or N-type layers, a series link of four P-type layers with different thicknesses and widths were used to assess the impact of thickness and width layer on the output of the power in the active layer (Figure 2a). Generally, when the amount of active layer was increased from one strip to four strips, the open circuit voltage increased proportionally for all of the active layers, and the same was true for the electric current output. As reported by Kwon, increasing the pair of the P-type Bi_0.4_Sb_1.6_Te_3_ and N-type Bi_2_Te_3_ layer grown by the metal organic vapor phase deposition can boost the power output [36]. Interestingly, the 6 mm wide and 40 mm long P-type layer (P4K-6mm) outperforms the thicker layer on Kapton for sample P45k-6 mm and a wide layer of 12 mm for P4K-12 mm in all of the strips as shown in Figure 10. In the meantime, the entire P-type layer has higher power output than the N-type layer on the Kapton substrate, i.e., N4k-6 mm.

## 4. Conclusions

The BiTe-based N-type and P-type thin films can be easily sputter deposited with a small target current on silicon, glass, and Kapton HN polyimide foil substrate using the DC magnetron sputtering mode. Fine columnar nano-sized grains were formed with a mixture phase of Bi_2_Te_3_ and Bi_6_Te_7.2_Se_1.8_ in the N-type thin film and a mixture of BiTe and Sb_2_Te_3_ in the P-type layer.

For all of the thin films, the thermal conductivity increased with the temperature. Generally, N-type layers have higher thermal conductivity and electrical conductivity in comparison with P-type layers, and both of the conductivity values have a trend of increase with elevated temperature, especially at a temperature above 0 °C. The Seebeck coefficient of P-type layers peaked at about 140 °C and was superior to that of the N-type layer, which grew steadily with temperature, while the N-type layer performed better at higher temperatures. The calculated ZT value has a similar trend to the Seebeck coefficient.

Power output tests on the different combinations of active layers suggest that a series link of active layers can maximize the output power. A series of four active layers suggests that the P-type layer has higher power output than the N-type layer on the Kapton substrate, and the longer the layer, the higher the output of current and the voltage. However, widening the strip or thickening the active layer did not improve the power output. Based on these findings, we will be able to design a device with optimized patterned thin-film strips on a flexible substrate to harvest the heat at a lower temperature range such as the photovoltaic solar cell for increasing efficiency.

## Figures and Tables

**Table 1:** Thermoelectric films produced by magnetron-sputtering techniques.

TE Film	Substrate	MS Parameters	Properties	Ref.
Sb_2_Te_3_	Polyimide	RF	TPF: 1.07 × 10^−4^ Wm^−1^K^−2^	[12]
Sb_2_Te_3_	Polyimide	DC, deposited at 473 K	TPF: 6.0 × 10^−4^ Wm^−1^K^−2^	[13]
Bi_2_Te_3_	SiO_2_/Si	RF	TPF: 3 × 10^−4^Wm^−1^K^−2^ at 498 K	[14]
Bi_2_Te_3_	Glass, alumina, sapphire, and polyimide	DC	S: maximum −1.63 × 10^−4^W/K on glass	[15]
Bi_2_Te_3_	Polyimide	RF/DC	TPF: 3.5 × 10^−3^ Wm^−1^K^−2^ at 558 K with DC power	[16]
Sb_2_Te_3_ Bi_2_Te_3_	Glass	RF	TPF: 1.27 × 10^−3^ Wm^−1^K^−2^ for Sb_2_Te_3_ at RT, and 1.02 × 10^−3^ Wm^−1^K^−2^ for Bi_2_Te_3_ at 573 K	[17]
Ag–Sb–Te (AST)	Soda-lime glass and SiO_2_/Si	DC	SiO_2_/Si wafer has better results with annealing at 773 K	[18]
Ge_2_Sb_2_Te_5_	Soda-lime Glass (523 K–723 K)	Pulsed DC	TPF: 0.77 × 10^−3^ Wm^−1^K^−2^ at 673 K	[19]
Zn_x_Sb_y_	flexible polyimide	RF	TPF: 2.35 × 10^−3^ Wm^−1^K^−2^	[20]
ScN	MgO (1123 K)	DC in Ar/N_2_	TPF: 3.3 × 10^−3^ Wm^−1^K^−2^ at 800 K	[21]
Cu_2_Se	Copper (low-temperature)	Pulsed hybrid technique	TPF: 1.1 × 10^−3^ Wm^−1^K^−2^ κ = 0.8 ± 0.1 Wm^−1^K^−1^	[22]
SnSe	Glass/fused silica	RF	TPF: 1.4 × 10^−4^ Wm^−1^K^−2^ at 575 K	[23,24]
Mg_2_Sn	Glass	RF	TPF: 8.5 × 10^−4^ Wm^−1^K^−2^ at 519 K	[25]
Mg_2_Si	Polyimide	RF	TPF: 3.3 × 10^−5^ Wm^−1^K^−2^ at 710 K	[26]
Ge–Au	Glass	Power of 320 W	Thermal conductivity: 1.1 Wm^−1^K^−1^ at 300 K	[27]
Zn_1−x_Ag_x_Sb	Fused silica	DC	TPF: 1.49 × 10^−3^ Wm^−1^K^−2^ at525 K	[28]
Amorphous Ga–Sn–O	Quartz	RF	TPF: 1.47 × 10^−4^ Wm^−1^K^−2^ at 397 K	[29]
Al-doped Zinc Oxide (AZO)	Soda-lime glass	RF	Maximum ZT value of 0.019 at 640 K	[30]
Co_x_Sb_y_	Polyimide	RF	Maximum TPF: 1.71 × 10^−4^ Wm^−1^K^−2^	[31]

DC: direct current, RF: radio frequency, TPF: thermoelectric power factor, PF: power factor, S: Seebeck coefficient, and κ: thermal conductivity.

**Table 2:** Sample codes and deposition details.

Sample Code	Substrate	Target Current (A)	Deposition Time (min)	Thickness (µm)
N4	Si/Chip/Glass	0.4	40	2.0
N5	Si/Chip/Glass	0.5	40	2.5
N45	Si/Chip/Glass	0.45	40	2.2
P3	Si/Glass	0.3	65	2.0
P3R	Si/Chip/Glass	0.3	50	1.5
P4	Si/Chip/Glass	0.4	50	1.8
P45	Si/Chip/Glass	0.45	45	1.8
P5	Si/Glass	0.5	45	1.9
P6	Si/Glass	0.6	30	1.8
P45K	Kapton	0.45	60	2.5
P4K-6mm	Kapton	0.4	50	2.0
P4K-12mm	Kapton	0.4	50	2.0
N4K-50m	Kapton	0.4	50	2.4

N3-S, N3-G, and N3-K indicate N3 thin film on silicon, glass, and Kapton substrate separately.

## Data Availability

Not applicable.

## Abbreviations

ALD	Atomic layer deposition
CVD	Chemical vapor deposition
DC	Direct current
EDX	Energy dispersive X-ray spectroscopy
MBE	Molecular-beam epitaxy
MOCVD	Metalorganic chemical vapor deposition
PF	Power factor
RF	Radio frequency
SEM	Scanning electron microscope
TE	Thermoelectric
TPF	Thermoelectric power factor
TEM	Transition electron microscope
XRD	X-ray diffraction
ZT	Figure of merit
